# Do Rats Have Epicardial Adipose Tissue?

**DOI:** 10.3390/biomedicines13071772

**Published:** 2025-07-20

**Authors:** Magdalena Kleszczewska, Katarzyna Czarzasta, Liana Puchalska, Łukasz Koperski, Agnieszka Cudnoch-Jędrzejewska, Małgorzata Wojciechowska

**Affiliations:** 1 Laboratory of Centre for Preclinical Research, Department of Experimental and Clinical Physiology, Medical University of Warsaw, 02-097 Warsaw, Poland; magdalena.kleszczewska@wum.edu.pl (M.K.); katarzyna.czarzasta@wum.edu.pl (K.C.); liana.puchalska@wum.edu.pl (L.P.); agnieszka.cudnoch-jedrzejewska@wum.edu.pl (A.C.-J.); 2 First Chair and Department of Cardiology, Medical University of Warsaw, 02-097 Warsaw, Poland; 3 Department of Pathology, Medical University of Warsaw, 02-097 Warsaw, Poland; lukasz.koperski@wum.edu.pl; 4 Invasive Cardiology Unit, Western Hospital, 05-825 Grodzisk Mazowiecki, Poland

**Keywords:** epicardial adipose tissue, fat depots, rodents, model organisms

## Abstract

The most frequently used laboratory animals for studies on adipose tissue properties and obesity are rodents. However, there are significant differences in the types of visceral fat depots between rodents and humans, including fat depots in the heart area. The large human fat depot of greatest interest in cardiac research is the epicardial adipose tissue (EAT). Its properties are widely investigated, because the EAT lies directly on the heart’s surface and can easily affect myocardial physiology. The major fat depot in rodents‘ chest—pericardial fat—is located on the ventral surface of the parietal lamina of the pericardium and is often incorrectly referred to as the EAT. Further confusion arises from reports claiming that rodents are entirely devoid of the EAT. We decided to verify adipose tissues in the heart area of 16 male Sprague Dawley rats under physiological conditions and in obesity. The animals in the NFD group (*n* = 8) were fed with a standard diet while these in the HFD group (*n* = 8) were fed with a high-fat diet (31% fat) starting from 4 weeks after birth. When the animals reached 12 weeks, the presence of fat deposits was verified. Additionally, their blood was collected to characterize carbohydrate and lipid metabolism changes, adipokine profile alterations, and their systemic inflammation status. The obesogenic diet caused significant disturbances in their carbohydrate and lipid metabolism, as well as hyperleptinemia. A high-fat diet primarily promoted the accumulation of pericardial fat, which was absent in the NFD rats and observed in 6 out of the 8 HFD animals. In both groups, adipocytes were also found directly on the hearts’ surfaces (EAT), albeit in very small numbers and limited to the atrioventricular groove on the dorsal side of the hearts. These adipocytes were dispersed among the vessels, making quantitative assessment and separation difficult, however, macroscopic evaluation revealed no noticeable differences in its extent. In conclusion, although rats are not entirely devoid of the EAT, their suitability for studying the properties of the EAT appears to be considerably limited.

## 1. Introduction

In response to the global obesity pandemic and its associated complications, particularly cardiovascular diseases, the adipose tissue has emerged as a major focus of scientific investigation in recent years. The epicardial adipose tissue (EAT) is of particular interest because of its direct contact with the myocardium and shared coronary microcirculation, which provides great opportunities for paracrine and endocrine influence [1]. Under physiological conditions in humans, the EAT covers approximately 80% of the heart’s surface, consistently surrounds the coronary arteries, and serves a protective function forthe heart [2]. It supplies cardiomyocytes with free fatty acids as a source of energy and in the case of their high concentrations in the coronary circulation, acts as a buffer, protecting cardiomyocytes against lipotoxicity [3]. Under pathological conditions, including obesity, the morphology and function of the EAT change. The EAT gets infiltrated by inflammatory cells and the secretion of pro-inflammatory cytokines increases. Subsequently, fibrosis occurs. Due to the increase in the amount of extracellular matrix, hyperplasia and hypertrophy of the adipocytes, and often insufficient angiogenesis, the density of the vascularization of fat deposits decreases. Hypoxia, which is a consequence of ischemia, increases the dysfunction and promotes death of the adipocytes [4,5]. These all change the metabolism of fats and carbohydrates in the EAT and the profile of secreted adipokines. Finally, the EAT gains pro-atherosclerotic and pro-arrhythmic properties and may contribute to the development of heart failure [6,7,8,9,10,11,12]. Similar changes occur in other fat depots, although their severity may vary.

The earliest discovered and most well-known adipokines include adiponectin and leptin. Experimental studies have demonstrated that adiponectin exerts numerous beneficial effects on the cardiovascular system. It counteracts conditions such as hypertension [13,14], myocardial hypertrophy [15] and atherosclerosis, primarily due to its anti-inflammatory properties [16,17,18,19]. Adiponectin is one of the few adipokines whose secretion decreases when the amount of adipose tissue increases [20]. The reduction in plasma adiponectin concentration in obesity is believed to result from adipocyte dysfunction and high local concentrations of pro-inflammatory cytokines, which inhibit its secretion [21,22]. Adiponectin is supposed to protect against the development of the metabolic complications of obesity, particularly insulin resistance [23,24].

The basic biological role of leptin is to signal sufficient energy supply in the body, which corresponds to the mass of adipose tissue [25,26]. In the hypothalamus, high concentrations of leptin produce an anorexic effect and increase energy expenditure (by stimulating the hypothalamic–pituitary–thyroid and hypothalamic–pituitary–gonadal axes) [27,28]. High leptin levels found in obese people (in simple obesity) do not cause a decrease in appetite, which suggests leptin resistance [29]. Leptin is considered a pro-inflammatory adipokine. It has been proven that leptin directly or indirectly affects immune system cells (neutrophils, T lymphocytes, monocytes/macrophages, and cytotoxic natural killer cells) increasing their proliferation, promoting their activation, and increasing the production of pro-inflammatory cytokines and free oxygen radicals [30,31,32,33,34]. Leptin is postulated to participate in the development of atherosclerosis, hypertension, and cardiac hypertrophy [35,36,37,38,39].

In the course of obesity, chronic low-grade systemic inflammation develops [40,41]. It has been suggested that a different profile of adipokines and cytokines, secreted by the adipocytes and immune system cells accumulating in fat deposits, is crucial for its occurrence [40,42,43,44]. Namely, in obesity, adipose tissue deposits release more pro-inflammatory cytokines and adipokines, including leptin and tumor necrosis factor alpha (TNF-α). This is not accompanied by the increase in anti-inflammatory cytokine secretion, including adiponectin and interleukin 10 (IL-10), needed to maintain balance [45]. On the contrary, in obesity, hypoadiponectinemia and often a decrease in plasma IL-10 levels are observed [46]. The chronic systemic inflammation that accompanies obesity contributes to an increased insulin resistance [47,48,49,50] and the development of other complications, including cardiovascular ones [51,52].

The most frequently used laboratory animals for obesity research are rodents. Obesity may be induced in rats or mice by high-fat or high-carbohydrate diets. Such models satisfactorily reflect the pathophysiology of obesity in humans (influence of the diet, coexisting carbohydrate and lipid metabolism disorders, hormonal changes, and systemic inflammation) [53].

However, it should be emphasized that there are significant differences in the types of visceral fat depots between rodents and humans. For example, omental fat, which is the major intra-abdominal visceral depot in humans, is poorly expressed in rodents. Similarly, the largest intra-abdominal depot in rodents, perigonadal fat, is not found in humans [54]. Some assume that perigonadal fat is functionally equivalent to human omental fat [54], but this assumption has its limitations. For example, blood from the perigonadal depot in rodents does not enter the portal circulation, unlike blood from the human omental depot [38]. Fat depots in the heart area of humans and rodents also differ. The types of cardiac depots in humans and their most commonly used definitions are shown in Table 1.

It should be emphasized that in the literature, there are often inconsistencies in the nomenclature of fat depots in the heart area. Some, for example, use the term “pericardial fat” to describe the pericardial and epicardial fat together. These are not depots with the same properties as the epicardial fat is separated from the pericardial fat by the visceral lamina of the pericardium [1]. In rodents, the primary fat depot is situated on the ventral side of the parietal pericardial lamina and is typically referred to as pericardial fat. However, it is sometimes inaccurately identified as the EAT [55,56,57]. Interestingly, some studies explicitly state that rodents are completely devoid of the EAT. We decided to verify and illustrate the presence of fat deposits in the heart area of 16 male Sprague Dawley rats under physiological conditions and in obesity. Additionally, we aimed to characterize their carbohydrate and lipid metabolism changes, adipokines profile alterations, and systemic inflammation status after 8 weeks of obesogenic diet.

## 2. Materials and Methods

The study was approved by the Second Local Animal Research Ethics Committee in Warsaw (WAW2/023/2019) and was consistent with the Directive 2010/63/EU of the European Parliament and of the Council of 22 September 2010 on the protection of animals used for scientific purposes. The ARRIVE guidelines were followed throughout the experimental procedures.

We examined 16 male Sprague Dawley rats bred in the Central Laboratory of Experimental Animals at the Medical University of Warsaw. At the age of 4 weeks, the rats were divided into two groups. The animals in the first group were fed with a standard diet (normal fat diet, NFD, containing 3.6% fat, 17.4% protein, and 60% carbohydrates; 2864 kcal/kg; Labofeed B, Kcynia, Poland; *n* = 8). The animals in the second group were fed with a high-fat diet (HFD, containing 31% fat, 17.1% protein, and 35.5% carbohydrates; 3842 kcal/kg; produced based on the standard of Labofeed B, Kcynia, Poland; *n* = 8). Food (as described above) and water were fed to the animals ad libitum. The animals were kept under 12 h/12 h light–dark cycle, in a standard Euro Type IV cage (1354G; Tecniplast S.P.A.; Buguggiate, Italy), with three rats per cage, in rooms equipped with mechanical ventilation, a controlled temperature (21–23 °C), and humidity at ±60% relative humidity.

All animals were examined at the age of 12 weeks, after a night fast. The animals were weighed and general anesthesia was administered (Ketamine 10 mg/100 g b.w. and Xylazine 1 mg/100 g b.w. intraperitoneally). Following a thoracotomy, the presence of the EAT was assessed macroscopically. A total of 2 mL of blood was collected from the internal jugular vein into tubes with EDTA- K2 anticoagulant and serum-separating tubes for biochemical analyses. Subsequently, one animal from each group was euthanized through the intraperitoneal administration of Morbital (Biowet, Puławy, Poland; 267 mg/kg b.w. penthobarbital sodium + 53.4 mg/kg b.w. penthobarbital), after which their hearts were excised. The remaining animals were allocated to other experimental protocols. The hearts were fixed in a 4% formaldehyde solution. The fixed samples were embedded in paraffin, cut into 4–5 μm sections, and stained routinely with hematoxylin and eosin for morphological examination. Images of the myocardium were captured with the Hamamatsu C9600-12 scanner using the NDP.scan software (v2.5.88; Hamamatsu, Hamamatsu City, Japan). The serum level of glucose, insulin, total cholesterol, high-density lipoprotein cholesterol, low-density lipoprotein cholesterol, and triglycerides were diagnosed by the Animal Diagnostic Laboratory (ALAB Weterynaria, Warsaw and Poznań, Poland). The insulin, adiponectin, leptin, IL-10, TNF-α level in plasma were determined using ELISA immunoassay kits (Rat/Mouse Insulin ELISA, EMD Millipore (Burlington, MA, USA); Rat Adiponectin ELISA, E091-R, Mediagnost; Mouse-/Rat-Leptin ELISA, E06, DRG MedTek (Warszawa, Poland); Rat (IL-10) ELISA Kit, SRB-T-83478, Shanghai Sunred Biological Technology Co., Ltd., Shanghai, China; Rat (TNF-α) ELISA Kit, SRB-T-82883, Shanghai Sunred Biological Technology Co., Ltd.). The tests were performed in accordance with the manufacturers’ instructions. A statistical analysis was performed using the STATISTICA program. The normality of the distribution of the variables was verified with the Shapiro–Wilk test. Comparisons between the two groups were made using Student’s *t*-test for normally distributed variables and the Mann–Whitney U test for non-normally distributed variables. The differences were considered significant at a significance level of less than 0.05.

## 3. Results

The HFD rats had statistically significantly higher serum concentrations of glucose (356 ± 23 mg/dL vs. 217 ± 16 mg/dL, *p* < 0.05), total cholesterol (80.0 ± 2.2 mg/dL vs. 65.0 ± 1.4 mg/dL, *p* < 0.05), triglycerides (134.0 ± 10.4 mg/dL vs. 61.0 ± 8.1 mg/dL, *p* < 0.05), and high-density lipoprotein cholesterol (62.0 ± 2.7 mg/dL vs. 51.0 ± 1.5 mg/dL, *p* < 0.05) as well as a higher plasma concentration of leptin (83 ± 36 pg/mL vs. 13 ± 3 pg/mL, *p* < 0.05) than the NFD rats. However, no significant differences were found in terms of insulinemia (HFD vs. NFD: 4.8 ± 0.9 pmol/L vs. 3.7 ± 0.4 pmol/L), the homeostasis model assessment of insulin resistance (0.6 ± 0.1 vs. 0.3 ± 0.02), and the serum concentrations of low-density lipoprotein cholesterol (12.2 ± 0.9 mg/dl vs. 12.7 ± 0.3 mg/dl), plasma adiponectin (3.6 ± 0.3 ng/mL vs. 3.9 ± 0.5 ng/mL), TNF-α (177 ± 2 pg/mL vs. 151 ± 7 pg/mL), and IL-10 (117 ± 7 pg/mL vs. 102 ± 10 pg/mL). Moreover, no significant difference in body weight was noted between both groups (HFD vs. NFD: 308 ± 6 g vs. 320 ± 3 g).

Following the thoracotomy, no EAT was macroscopically visible on the ventral surface of any heart. A distinct accumulation of adipocytes was observed in this cardiac region; however, it was located on the parietal lamina of the pericardium and therefore classified as a pericardial adipose tissue. Pericardial fat was present in six out of the eight high-fat diet (HFD) rats but was absent in all the normal-fat diet (NFD) rats.

**Figure 1:**
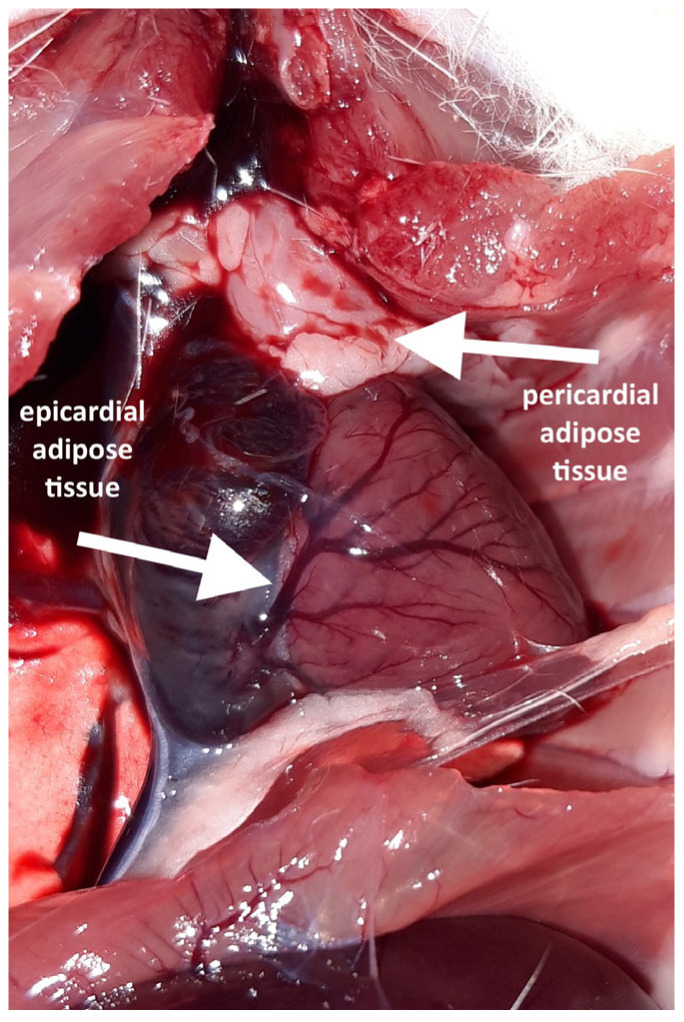
Fat depots visible after opening the chest and rotating the heart.

**Figure 2:**
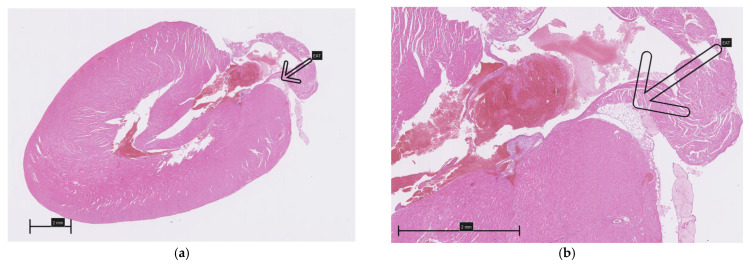

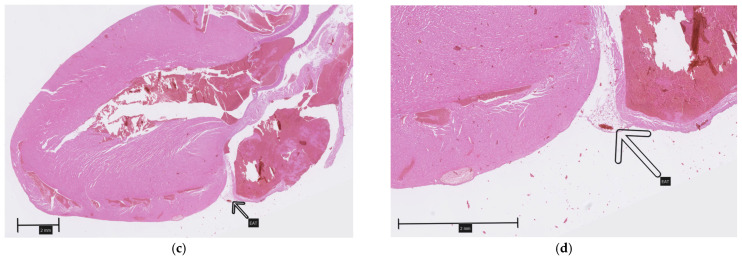
Epicardial adipose tissue located on the dorsal surface of the hearts within the atrioventricular groove (arrows): (**a**,**b**)—HFD; (**c**,**d**)—NFD.

A careful inspection of the hearts revealed a small amount of epicardial adipose tissue forming a thin, pale band located directly on the dorsal surface of the hearts, within the atrioventricular groove (Figure 1). A histopathological examination of both collected hearts (one from each group) confirmed the presence of fat cells in this localization, which were dispersed among blood vessels (Figure 2). Although no direct quantification was performed, a visual assessment suggested no significant difference in the amount of EAT between the HFD and NFD rats.

## 4. Discussion

The result of our observation is consistent with the reports of other scientists who verified the presence of the EAT in mice and rats [58]. Therefore, it is not true that rodents are completely devoid of the EAT, but because the EAT is present only in a specific location and in small amounts, the possibilities of studying its properties in classic animal models (rodents) are very limited. Perhaps in the aforementioned publications [56,57], the EAT is actually a pericardial tissue, just incorrectly named. Experts emphasize the problem of incorrect naming, which may lead to confusing conclusions [59]. However, the pericardial adipose tissue in rodents is similar to the EAT in humans in terms of its secretory activity [60]. Conducting research to examine the influence of visceral fat on the heart and coronary vessels in rodent models may be justified by the fact that there are multiple pores in the pericardial laminas in rat and mouse hearts. These pores enable communication between the pericardial adipose tissue and myocardium, including the influence of adipokines on the heart [61].

Cinti et al. proved that all fat depots constitute a continuum (they are anatomically connected) [62]. The lack of a pericardial adipose tissue on the ventral surface of the heart in some rats that we studied can be explained by the fact that the fat depot in the chest was smaller, covering only the para-aortic region and not passing to the ventral surface of the heart.

Despite their anatomical connection, fat depots differ in their cellular composition (including the number of immune cells physiologically present in the adipose tissue), rate of adipogenesis, cell senescence and apoptosis, and immunological, endocrine, and metabolic activities [54]. Most data are concerning the differences between visceral and subcutaneous deposits. For example, it has been shown that the visceral adipose tissue is metabolically and secretionally more active than the subcutaneous adipose tissue [43]. In obesity, the visceral adipose tissue is characterized by a greater expression of pro-inflammatory cytokines, which, at least in part, results from a more intense influx of macrophages compared to the subcutaneous adipose tissue [63,64,65]. Beyond the well-established differences between the visceral and subcutaneous adipose tissue, distinct characteristics have also been identified among various visceral fat depots. For example, the EAT differs from other visceral deposits in terms of a smaller adipocyte size, a higher protein content, a more intensive uptake of free fatty acids, a higher rate of insulin-dependent lipogenesis, lower glucose consumption, and less reduction during starvation [66].

In the study, no significant differences in body weight were found between the HFD and NFD rats; however, animals fed a high-fat diet were characterized by significant biochemical changes typical of obese animals (hyperglycemia, dyslipidemia, and hyperleptinemia). Obesity in humans can be diagnosed not only on the basis of an increased body mass index (taking into account body weight and height), but also on the basis of increased fat content in the body. There is no definition of obesity in animals [50]. Most researchers consider animals from experimental groups (fed with an appropriate diet) that differ significantly in body weight or body fat content from control groups as obese [53]. It is even suggested that measuring body fat is a more sensitive criterion for assessing obesity. In one study, in rats fed for 10 weeks with a high-fat diet in which fat accounted for 40% of its calorie content, only a 10% increase in body weight was accompanied by as much as a 40% increase in body fat content compared with animals that were fed with a low-fat diet [67].

Obesity, both in animals and in humans, is typically accompanied by hyperleptinemia and hypoadiponectinemia. In the experiment, the HFD rats had a higher plasma leptin concentration and lower plasma adiponectin concentration than the NFD rats, but statistical significance was achieved only in the case of leptin. This may mean that significant changes in adiponectin secretion occur more slowly than in the case of leptin. The plasma concentrations of TNF-α and IL-10 did not differ statistically significantly between both groups, which may suggest that the 8-week obesogenic diet did not lead to the development of systemic inflammation. Currently, it is being pointed out that inflammation in local fat deposits (for instance around the heart and blood vessels) may be more important for the development of obesity complications than inflammation at the systemic level [68,69,70].

## 5. Limitations of the Study

This study has several limitations, including a small sample size and the exclusion of female rats. While generalizing conclusions regarding the presence of the EAT in females is not supported by our data, such extrapolation may be cautiously justified based on findings reported by other authors [58].

Furthermore, we did not perform an exact quantification of the EAT and are therefore unable to compare the EAT volume between the HFD and NFD animals. The EAT was found as a thin, pale band located directly on the dorsal surface of the hearts—histopathological analysis was performed only in two animals; however, it was used solely to confirm the presence of fat cells in this localization. Future studies employing cardiac computed tomography could allow for more precise measurements of the EAT, and using a higher-fat diet over a longer period might reveal potential differences in the EAT volume. Finally, it would be valuable to extend the study to include additional time points in order to assess potential changes in body weight, hypoadiponectinemia, and systemic inflammation. Moreover, investigating the concentrations of inflammatory cytokines directly within adipose tissue depots would provide further insight into their local inflammatory status.

## 6. Conclusions

Our findings indicate that in Sprague Dawley rats, a high-fat diet primarily promotes the accumulation of pericardial fat. Although rats are not entirely devoid of epicardial adipose tissue, it is confined to a specific location and present in small amounts. This prompted us to address a broader issue: the frequent misidentification of pericardial fat as the EAT in rodent studies. We conclude that inconsistent terminology contributes to confusion and that rats may not be a suitable model for studying the human EAT.

Additionally, an obesogenic diet rapidly induces disturbances in carbohydrate and lipid metabolism, as well as hyperleptinemia, even before significant weight gain. In contrast, decreased adiponectin levels and inflammation likely occur at later stages.

## Figures and Tables

**Table 1:** Types of fat depots in the heart area of humans and their most commonly used definitions.

The Type of Fat Depot	Definition
epicardial	adipose tissue between the myocardium and the visceral lamina of the pericardium
pericardial	adipose tissue between the visceral and parietal lamina of the pericardium and fat on the outer surface of the parietal lamina of the pericardium
paracardial	adipose tissue outside the pericardium, sometimes called the mediastinal depot
perivascular	adipose tissue surrounding blood vessels, including coronary vessels
ectopic	triglyceride depots within cells and tissues other than the adipose tissue (e.g., in cardiomyocytes)

## Data Availability

Data are contained within the article.

## Abbreviations

The following abbreviations are used in this manuscript:

EAT	Epicardial adipose tissue
TNF-α	Tumor necrosis factor alpha
IL-10	Interleukin 10
